# Organizational Support and Reward Systems as Drivers of Teleworker Engagement: The Mediating Role of Organizational Trust

**DOI:** 10.3390/bs16071183

**Published:** 2026-07-13

**Authors:** Elisabeth Figueiredo, Clara Margaça, José Carlos Sánchez-García, Paulo Almeida Pereira

**Affiliations:** 1 Department of Social Psychology and Anthropology, Faculty of Psychology, University of Salamanca, 37005 Salamanca, Spain; efigueiredo@usal.es (E.F.); jsanchez@usal.es (J.C.S.-G.); 2 Instituto de Gestāo e das Organizações da Saúde, Universidade Católica Portuguesa, 3504-505 Viseu, Portugal; ppereira@ucp.pt

**Keywords:** telework, reward systems, perception of organizational support, organizational trust, work engagement

## Abstract

The adoption of telework has intensified the need to understand how organizational practices influence employee behaviors in contexts characterized by reduced face-to-face interaction. Based on Social Exchange Theory, this study examines the effects of perceived organizational support and reward systems on work engagement among teleworkers and explores the mediating role of organizational trust. A cross-sectional quantitative design was employed, using survey data collected from 405 teleworkers operating in Portugal. The data were analyzed using structural equation modeling to test the proposed relationships. The results indicated that both perceived organizational support and reward systems exert statistically validated positive effects on work engagement, with reward systems being the strongest predictor. Organizational trust played a mediating role, reinforcing the impact of support and rewards on engagement. These results suggested that organizational practices function not only as instrumental resources but also as relational signals that shape trust and promote reciprocal employee responses in remote work contexts. By integrating support, rewards, trust, and engagement into a single analytical framework, this study contributed to the literature on telework and human resource management. The results reinforce the importance of management practices rooted in trust and support to promote engagement and organizational effectiveness in remote work environments.

## 1. Introduction

Over the past few decades, the world of work has undergone significant transformations, some of which were accelerated by the COVID-19 pandemic (Balgiu, 2023; Nemțeanu et al., 2022). One of the most notable changes was the increase in teleworking, which brought new challenges for companies and workers (Pass & Ridgway, 2022; Thevenon, 2021). Telework refers to work performed remotely using ICT, outside organizational premises (Figueiredo et al., 2021). While teleworking is not a new phenomenon (Brunelle & Fortin, 2021; Gohoungodji et al., 2023), it became widespread in March 2020 with the COVID-19 outbreak (Berberat et al., 2021; Cianferoni, 2021; OIT, 2020). This new way of working has shown that workers can be equally productive when performing their duties remotely (Bernatchez, 2022; Brunelle & Fortin, 2021). Thus, its increasing adoption was visible (Brunelle & Fortin, 2021), encompassing more sectors and employees (Bernatchez, 2022) and requiring regulation (Eurofund, 2022; Carvalho, 2022) due to its repercussions on organizational dynamics.

The increasing value placed on teleworking (e.g., Balgiu, 2023; Bernatchez, 2022; Gohoungodji et al., 2023; Sanhokwe, 2022) suggests that it should be seen as a success factor in process management of companies. However, despite the various advantages of teleworking (e.g., flexibility, time and money savings on commuting) (Figueiredo et al., 2021), it also entails a set of challenges and consequences at the individual and business levels. This paradigm shift calls organizations to invest in a culture of engagement, aiming to motivate teams and establish a mutual commitment (Berberat et al., 2021). The literature highlights support and reward policies as motivational factors and strategic tools that influence employee behavior (Gandrita et al., 2025), increasing job satisfaction, trust, and commitment to organizational goals (Paillé et al., 2015; Pass & Ridgway, 2022; Sanhokwe, 2022). Hence, it is crucial to understand the new dynamics of the world of work to better deal with the resulting challenges (Berberat et al., 2021; Cianferoni, 2021; Thevenon, 2021), especially since there is a large segment of workers who wish to remain in telework (Pass & Ridgway, 2022; Sanhokwe, 2022).

Perceived organizational support is considered a success factor in a telework context (Gohoungodji et al., 2023; Sanhokwe, 2022). This construct encompasses perceptions of the support, including both resources and tools for performing tasks, as well as social and emotional support. Adequate organizational support can positively influence workers’ trust in the organization and their level of engagement (Kurtessis et al., 2017; Paillé et al., 2015; Mehmood et al., 2023). Reward systems, which can take the form of financial or non-financial incentives, have been considered efficient resources with positive impacts on employee behavior (Figueiredo et al., 2024; Kamselem et al., 2022). Implementing an effective reward system aligned with organizational goals can motivate remote workers to achieve better results, strengthening trust in the organization and greater commitment (Figueiredo et al., 2025; Hoole & Hotz, 2016; Ji & Cui, 2021).

Previous research in traditional work environments has consistently demonstrated that organizational trust is an important predictor of employee engagement, suggesting that workers who perceive higher levels of trust show greater dedication and involvement (Dirks & Ferrin, 2002; Kramer & Tyler, 1996). Authors such as Paillé et al. (2015) and Vanhala and Dietz (2019) have conceptualized trust as a dynamic perception that develops over time through employees’ experiences with organizational trust and support, shaping, and sustaining work engagement. Based on these findings, research has also examined this relationship in telework settings, where employees work more independently and have less face-to-face contact. For example, Hussain et al. (2026), Mehmood et al. (2023), and Utomo et al. (2023) have reported a strong positive association between these two constructs, indicating that organizational trust remains a central factor in explaining worker engagement in telework contexts. Although its development appears to depend heavily on the quality of communication, leadership visibility, and employees’ perceptions of the organization’s responsiveness (Ha & Lee, 2022; Sipayung & Nilasari, 2026), existing literature suggests that perceived organizational support and fair reward systems are equally fundamental. Thus, when employees perceive that the organization values their well-being (Mehmood et al., 2023) and ensures fairness in recognition and rewards, trust is more likely to emerge and be maintained (Sipayung & Nilasari, 2026).

In this study, perceived organizational support and reward systems are conceptualized as critical organizational resources (Utomo et al., 2023) that shape how employees interpret their relationship with the organization and develop trust in it, which, in turn, shapes their attitudes, behaviors, and level of engagement (Gohoungodji et al., 2023; Serhan et al., 2026). Based on this perspective, the study aims to analyze the relationships between perceived organizational support (POS), reward systems (RS), organizational trust (OT), and work engagement (WE). More specifically, the research explores: (1) the direct effects of perceived organizational support and reward systems on work engagement; (2) the influence of perceived organizational support and reward systems on organizational trust; and (3) the mediating role of organizational trust in the relationship between perceived organizational support, reward systems, and work engagement.

While previous studies (e.g., Sanhokwe, 2022) have explored the interaction between these variables in in-person work contexts, a research gap remains in our understanding of how these relationships function in teleworking. In particular, the mediating role of organizational trust in the relationship between organizational practices and work engagement remains insufficiently explored in remote work environments (Hussain et al., 2026; Wawrzynek, 2023). By integrating these organizational dimensions, this approach provides a comprehensive understanding of how organizational factors interact to influence employee engagement in teleworking contexts. This theoretical framework integrates all variables into a single conceptual model, in which organizational trust mediates the relationship between organizational practices and employee engagement at work.

Regarding the theoretical assumptions, this study is grounded on the Social Exchange Theory (SET), initially proposed by Homans (1958) and later developed by Kieserling (2019) and Emerson (1976). According to SET, social relations are based on the exchange of resources, both tangible and intangible, in which individuals seek to maximize benefits and minimize costs. The theory also emphasizes the importance of reciprocity, mutual obligations, trust (Kieserling, 2019), dependence, and interdependence in the formation of social interactions and the maintenance of relationships over time (Emerson, 1976). In the organizational context, SET explains how employees’ behavior is shaped by the evaluation of the benefits and costs resulting from their interactions with the organization. In this sense, when they perceive that the organization values their contribution and offers adequate support and fair rewards, they adopt positive behaviors, such as work engagement, organizational trust, and loyalty (Paillé et al., 2015).

In telework contexts, employees have fewer face-to-face interactions and may experience a weaker connection with the organization (Figueiredo et al., 2024), which becomes a particularly relevant subject of study. Hence, the application of Social Exchange Theory helps to elucidate the mechanisms by which organizational attributes are presumed to influence work engagement.

In a practical view, this study offers relevant implications for human resource management in teleworking contexts. The data highlights the need for organizations to promote adequate organizational support and implement effective reward systems. Moreover, organizational trust is considered a mediating variable in the interplay between support, rewards, and engagement. The article synthesizes the main findings, highlighting their theoretical and practical contributions, and suggests directions for future research.

## 2. Theoretical Framework and Hypotheses Development

### 2.1. Work Engagement

Engagement is a multidimensional construct (e.g., Hoole & Hotz, 2016; Schaufeli, 2014), and according to Schaufeli et al. (2002), it is “a positive, fulfilling, work-related state of mind that is characterized by vigor, dedication, and absorption” (p. 74). Advocated by Kahn in the 1990s (Kahn, 1990; Yadav et al., 2022), the concept of work engagement has received increasing interest not only in organizational contexts but also from scholars (Khusanova et al., 2021; Huang et al., 2022). According to Kaur and Randhawa (2020), workers who are diligently engaged in their tasks and responsibilities generally exhibit a sense of purpose and meaning in relation to their roles, feeling valued and recognized for their contributions (Yadav et al., 2022). The literature highlights engagement as a crucial organizational outcome, associating it with positive business outcomes and improved corporate performance (Ji & Cui, 2021; Yadav et al., 2022).

To optimize work engagement, factors such as access to information, skills development, autonomy, rewards, and recognition are fundamental (Swaroop & Dixit, 2022). Marodin et al. (2023) and Krishnaveni and Monica (2018) state that recognition is a powerful lever that fosters a climate of trust and contributes to a higher level of employee effort in performing their duties (Bilodeau et al., 2024).

### 2.2. Organizational Trust

Organizational trust also plays a crucial role in organizational effectiveness and performance (Kramer & Tyler, 1996). Organizational trust is a fundamental component of business efficiency, as it reinforces a constructive relationship between worker and company, contributing to positive human resource management (Yadav et al., 2022). Key factors such as transparent communication, leadership, fair business policies and practices (Ha & Lee, 2022), responsiveness to workers’ needs, and contractual compliance influence the building of trust (Vanhala & Dietz, 2019). For instance, Maslikha (2022) analyzed the relationship between organizational trust and work engagement, concluding that organizational trust can increase work engagement. Furthermore, the authors also found that engagement was a consequence of employees’ perception that the organization is trustworthy, honest, and competent.

### 2.3. Perception of Organizational Support and Reward Systems

Reward systems and the perception of organizational support have been extensively studied, aiming to uncover the effects of these practices on worker behavior (Paillé et al., 2015; Sweiss & Yamin, 2020). Some studies (e.g., Sweiss & Yamin, 2020) demonstrated the influence of organizational awareness on reshaping support and reward policies, meeting worker expectations, and reinforcing effective culture and management (Brunelle & Fortin, 2021; Ngamkroeckjoti et al., 2022).

Reward management is identified as a key factor in increasing employee productivity and engagement (Tarigan et al., 2022). Organizations seeking to increase worker output and optimize the level of execution of business tasks should also link reward policies (Beqiri & Aziri, 2022). According to Ngamkroeckjoti et al. (2022), extrinsic and intrinsic rewards are among the most significant factors in worker motivation. Empirical evidence (e.g., Leitão et al., 2022; Pajor & Szeberényi, 2025) demonstrates that reward systems influence teleworkers’ behavior, leading them to increase their efforts and improve the quality of work. However, given that these employees are outside the reach of hierarchical bodies (Pass & Ridgway, 2022), the likelihood of receiving immediate rewards and positive feedback on their performance may be lower compared to an in-person context (Brunelle & Fortin, 2021; Gohoungodji et al., 2023). Thus, a non-transparent and inadequate reward system can compromise work engagement and employee productivity (Nemțeanu et al., 2022).

### 2.4. Perception of Organizational Support and Organizational Trust

Organizational support refers to how an organization meets the needs and interests of its employees. According to Eisenberger et al. (1986), this concept concerns the employees’ perception of the company’s support measures, the value placed on their efforts, and the degree of care shown for their well-being and satisfaction (Sanhokwe, 2022), which directly contribute to performance and productivity.

Authors such as Kurtessis et al. (2017) affirm that this perception is understood as a variable that simultaneously builds and influences trust. Demirel and Yalman (2022) demonstrated a meaningfully positive relationship between perceived organizational support (POS) and organizational trust. These authors concluded that POS was one of the most determining factors in the formation of stable trust relationships between employees and the organization. Similarly, Paillé et al. (2015) highlighted that well-designed organizational support positively influences both organizational trust and employee engagement. Thus, when workers perceive that the organization supports them and considers their needs, they adopt a positive attitude and become more confident in their workplace (Bentley et al., 2016), contributing to business success (Hong et al., 2023; Paillé et al., 2015).

This relationship becomes even more relevant in remote work contexts. The reduced frequency of face-to-face interactions and the reliance on digital communication channels increase employees’ dependence on perceived organizational support to interpret organizational intentions and fairness (Sipayung & Nilasari, 2026). On the other hand, when organizations actively support employees working from home, they foster more stable relational conditions that sustain trust and engagement over time (Wawrzynek, 2023). Based on this, the following hypothesis is presented:

**Hypothesis** **1:** *There is a positive and statistically significant relationship between the perception of organizational support and organizational trust.*

### 2.5. Reward Systems and Organizational Trust

According to Yadav et al. (2022), many professionals seek companies that promote a positive work environment (Hong et al., 2023). As important as professional success, workers prioritize companies that meet their needs and interests and offer performance-based benefits (V. Santos et al., 2019). Valuing performance through a reward system (V. Santos et al., 2019) is a powerful management strategy that contributes to motivation (Saether, 2020), satisfaction, productivity (Tarigan et al., 2022), and organizational trust (Mehmood et al., 2023). Workers who work remotely become even more dependent on transparent and fair reward systems due to reduced physical proximity to supervisors, which increases the need for clear performance criteria and consistent evaluation practices (Mensah et al., 2024). Intrinsic and extrinsic rewards, as well as support measures, properly implemented and perceived by these employees, enable engagement (Pajor & Szeberényi, 2025), proactivity, creativity, and autonomy (Nemțeanu et al., 2022; Ngamkroeckjoti et al., 2022; Leitão et al., 2022). Based on this premise, the following hypothesis is raised:

**Hypothesis** **2:** *There is a positive and significant relationship between reward systems and organizational trust.*

### 2.6. Organizational Trust and Work Engagement

Mehmood et al. (2023) and Vanhala and Dietz (2019) assert that organizational trust influences employee behavior. Dirks and Ferrin (2002) concluded that organizational trust benefits both employees and the organization, improving productivity and organizational performance. However, authors such as Brunelle and Fortin (2021) stated that situations in which employees feel undervalued can cause demotivation and lack of confidence, compromising performance, commitment, and productivity. When employees perceive that the organization does not offer adequate support, organizational trust is compromised (Wawrzynek, 2023), and motivation and engagement are very low (Vanhala & Dietz, 2019). For remote workers, organizational trust plays a key role, reducing uncertainty and fostering employees’ confidence in the organization’s intentions and decisions (Wawrzynek, 2023). In turn, trust is positively associated with higher work engagement, leading remote employees to feel more involved, motivated, and absorbed in their work. Similarly, when employees working from home trust the organization they work for, they are more likely to perform their duties with greater commitment (Kaplan et al., 2018; Nemțeanu et al., 2022; Ngamkroeckjoti et al., 2022). Thus, the following hypothesis is raised:

**Hypothesis** **3:** *There is a positive and significant relationship between organizational trust and work engagement.*

### 2.7. Perception of Organizational Support and Work Engagement

The perception of organizational support (POS) plays a key role in promoting work engagement (Paillé et al., 2015), and it is associated with efficiency, motivation, and business commitment (Aldabbas et al., 2023; Mehmood et al., 2023; Bilodeau et al., 2024; Selander & Alasoini, 2025). According to Kurtessis et al. (2017), the support provided can contribute to the psychological well-being and generate job satisfaction. Thus, by demonstrating this concern, organizations trigger greater involvement in task completion (Swaroop & Dixit, 2022; Aldabbas et al., 2023) and a feeling of belonging (Bilodeau et al., 2024). Adequate support creates a breeding ground for motivated and productive employees aligned with organizational goals (Mascarenhas et al., 2022). In telework settings, higher levels of POS are associated with greater motivation, greater organizational trust, and greater work engagement (Mehmood et al., 2023). Therefore, the following hypothesis arises:

**Hypothesis** **4:** *There is a positive and significant relationship between the perception of organizational support and work engagement.*

### 2.8. Reward Systems and Work Engagement

Corporate reward systems are structures designed to recognize, reward, and encourage workforce performance and behavior. Rewards play an important role in employee motivation, influencing job satisfaction, engagement, and efficiency (Nemțeanu et al., 2022; Gandrita et al., 2025). Nemțeanu et al. (2022) concluded that effective reward management is crucial for organizational success and can significantly increase employee motivation and dedication in their duties (Hoole & Hotz, 2016). The results show that, in telework or hybrid work environments, reward systems are considered effective and positive resources that impact employee behavior. In this sense, a reward system can shape organizational behavior and promote trust, ensure better performance (Manzoor et al., 2021; Pajor & Szeberényi, 2025), and strengthen commitment (Figueiredo et al., 2025). Thus, the following hypothesis is presented:

**Hypothesis** **5:** *There is a positive and significant relationship between reward systems and work engagement.*

### 2.9. Conceptual Framework

**Figure 1:**
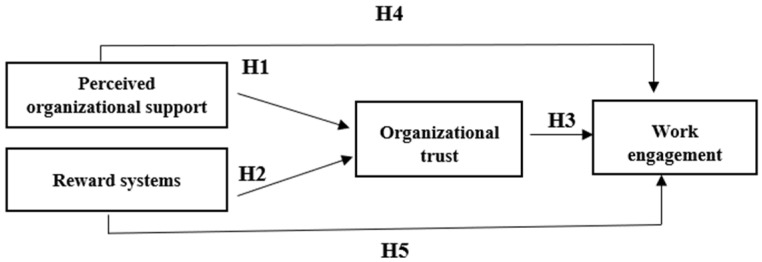
Conceptual model.

Based on the reviewed literature, the conceptual model presented in Figure 1 is grounded in the articulation of three central theoretical assumptions: (1) perceived organizational support and reward systems positively influence employees’ perception of the organization; (2) these organizational practices foster the development of organizational trust; and (3) organizational trust contributes to higher levels of work engagement. Furthermore, the model presupposes direct effects of perceived organizational support and reward systems on work engagement, as well as indirect effects mediated by organizational trust. Based on this theoretical framework, the hypotheses described above were formulated to examine the direct and indirect relationships investigated in this study.

## 3. Methods

### 3.1. Procedure

This study aims to investigate the impact of organizational support and reward systems on the work engagement of teleworkers, with a focus on the mediating role of organizational trust. To this end, a quantitative, descriptive, cross-sectional, and exploratory approach was adopted. Data collection, which took place between 1 March 2025 and 31 August 2025, was conducted using a questionnaire administered via Google Forms, sent by email to teleworkers from companies based in Portugal and disseminated on social media.

Participation was voluntary, confidential, and anonymous, with participants giving their informed consent before responding. No incentives, financial or otherwise, were offered for participation. All participants gave their free and informed consent before participating in the study. The study was conducted in accordance with the Declaration of Helsinki.

### 3.2. Participants

The convenience sample consists of 405 teleworkers residing in Portugal. Table 1 shows that the age ranged from 22 to 53 years (M = 31.87; SD = 9.43), with a predominance of female participants (55.3%). The majority held a master’s degree (52.3%). Regarding marital status, 61.7% were single. Regarding position, 68.4% of employees did not hold management positions and had between 1 and 5 years of professional experience (73.8%), and 3.7% had more than 10 years of seniority. For the majority of participants (87.2%), teleworking was determined by the company.

### 3.3. Instruments

A sociodemographic questionnaire and a set of four 5-point Likert-type scales were applied, ranging from 1 “strongly disagree” to 5 “strongly agree”. The study constructs were measured using previously validated scales. Portuguese versions of the instruments, with proven validity and psychometric properties, were used. Only one instrument (RS) required translation and cultural adaptation for the Portuguese context.

Perceived Organizational Support (POS), 8 items, was measured using the short version of the Perceived Organizational Support Scale (Eisenberger et al., 1986), which was translated and validated for the Portuguese population by J. Santos and Gonçalves (2010). In a recent Portuguese study, José et al. (2025) reported satisfactory fit indices and a very good Cronbach’s alpha for this scale (α = 0.91 for affective POS; and α = 0.87 for cognitive POS). It should be noted that 4 items were reversed (2, 3, 5, and 7).

Reward Systems (RS), 11 items, were measured using the short version of the Pay Satisfaction Questionnaire (Heneman & Schwab, 1985). For this purpose, the scale was translated into Portuguese, following the guidelines of the International Test Commission (2010) for the cross-cultural adaptation of psychological instruments, which involved independent translation and back-translation by bilingual experts, as well as verification of semantic equivalence with the original version.

Organizational trust (OT), 7 items (3, 5, and 7 reversed), was assessed using the Organizational Trust Scale developed by Gabarro and Athos (1976), adapted by Robinson and Rousseau (1994), which reported high internal consistency (α = 0.90), and translated and validated to Portuguese by Costa (2016), with good internal consistency (α = 0.82).

Work engagement (WE) with 9 items was measured using the reduced version of the Utrecht Work Engagement Scale (Schaufeli & Bakker, 2003), which was validated for the Portuguese population by Sinval et al. (2018) (α = 0.95). This scale comprises three dimensions: vigor (energy level and resistance to effort; items 1, 2, and 5), dedication (affective involvement and pride; items 3, 4, and 7), and absorption (level of concentration and total immersion in work; items 6, 8, and 9).

All factor loadings were adequate and above recommended thresholds (Table 2). Internal consistency was excellent for all constructs: POS (α = 0.887), RS (α = 0.908), OT (α = 0.942), and WE (α = 0.979), confirming strong internal consistency.

### 3.4. Statistical Analysis

The chi-square test (χ^2^), Pearson correlation, and t-student test were performed, and Cronbach’s alpha (α) was used to assess internal consistency. The Statistical Package for the Social Sciences (SPSS) software, version 30, was used. Following Hair et al. (2019) and considering that constructs’ validity depends on the characteristics of the sample and the context of application, a confirmatory factor analysis (CFA) was performed. Subsequently, to explore the relationships among the constructs and after verifying the normality assumption, IBM SPSS AMOS 26 was used for structural equation modeling (SEM) analysis, using the maximum likelihood estimation method (Marôco, 2018).

To assess the model fit quality (Figure 1), several indices were considered: the χ^2^/df ratio, the Comparative Fit Index (CFI), the Tucker–Lewis Index (TLI), the Incremental Fit Index (IFI), and the Root Mean Square Error of Approximation (RMSEA) (Hu & Bentler, 1999). Values of χ^2^/df < 3, CFI, IFI, and TLI > 0.90, and RMSEA < 0.08 indicate a good fit of the model to the data (Arbuckle, 2022). A *p*-value < 0.05 was considered statistically significant.

To complement the reliability analysis, convergent validity was also examined using the AVE, based on indicator loadings and critical ratios, and discriminant validity, considering correlations among factors (Hair et al., 2019). AVE and item loading values above 0.50 are considered satisfactory (Kline, 2016). Discriminant validity is confirmed when the observed correlations between the constructs are less than 0.85 (Marôco, 2018). The sample (*n* = 405) meets the proposed requirements for SEM, with an approximate statistical power of 0.90, ensuring consistency in the proposed structural relationships.

#### 3.4.1. Confirmatory Factor Analysis (CFA) and Cronbach’s Alpha

Table 2 shows the CFA results. The item factor loading values, ranging from 0.700 to 0.952, indicate high psychometric quality and suggest strong convergent validity (Fornell & Larcker, 1981), with all items showing a satisfactory association with their respective constructs. The α values support high internal consistency (Hair et al., 2019), ranging from 0.887 to 0.979.

#### 3.4.2. Construct Reliability and Validity

Composite reliability (CR) and AVE were assessed. As shown in Table 3, all constructs presented CR values greater than 0.90, indicating high internal consistency (Hair et al., 2019). AVE ranged from 0.526 to 0.862, suggesting good convergent validity, since all values exceeded the recommended threshold of 0.50 (Fornell & Larcker, 1981). Discriminant validity was confirmed, since the square root of the AVE of each construct is greater than the correlations between the remaining factors. These results indicate that, despite the relationships among the constructs, they remain distinct and represent different theoretical concepts, which reinforces the validity of the model (Fornell & Larcker, 1981).

## 4. Results

### 4.1. Descriptive Statistics and Correlation Analysis

Table 4 shows the descriptive analysis and correlations, presenting a moderate and homogeneous dispersion of the data. All correlations between the POS, RS, OT, and WE constructs were statistically significant.

### 4.2. Structural Equation Modeling

Table 5 presents the χ^2^/df ratio values and the RMSEA, CFI, TLI, and IFI fit indices. The results confirm the consistency of the proposed model (Arbuckle, 2022).

Table 6 shows that all structural pathways presented statistical significance, confirming the validity of the model. POS significantly and positively influences OT (β = 0.292, t = 5.084; *p* < 0.01), supporting H1. This result suggests that higher levels of POS lead to increased organizational trust (OT). Hypothesis H2 was also supported, showing that RS positively influences OT (β = 0.342, t = 5.939; *p* < 0.01). The results reveal that OT has a statistically validated positive effect on WE (β = 0.186, t = 3.313; *p* < 0.01), supporting H3. POS significantly affects WE (H4) (β = 0.393, t = 6.275; *p* < 0.01). Finally, hypothesis H5 was also supported, demonstrating that reward systems (RS) were the strongest predictor of work engagement (WE) (β = 0.806, t = 10.862, *p* < 0.01).

**Figure 2:**
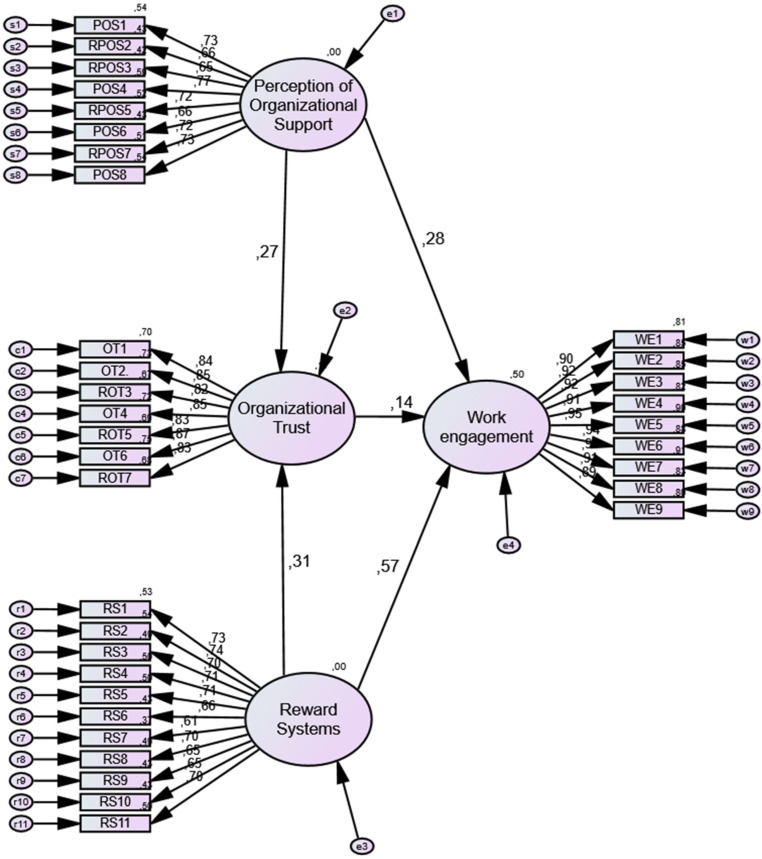
SEM results for mediating effects.

Additionally, Figure 2 presents the standardized coefficients of the model and the results obtained from SEM. The effect of POS on OT was significantly positive (β = 0.27; *p* < 0.01). The relationship between RS and OT was also statistically significant and positive (β = 0.31; *p* < 0.01). Similarly, the path between OT and WE showed a significant effect (β = 0.14; *p* < 0.01). The direct relationship between POS and WE was positive and significant (β = 0.28; *p* < 0.01), as was the relationship between RS and WE, which presented the highest coefficient in the model (β = 0.57; *p* < 0.01).

All hypothesized relationships in the SEM were supported, corroborating the central role of organizational trust as a mechanism connecting perceived organizational support and reward systems to work engagement. Furthermore, both perceived organizational support and reward systems exerted direct effects on work engagement, with reward systems being the strongest predictor in the model. These findings are consistent with the proposed theoretical framework and suggest that organizational practices aimed at strengthening employee support, trust, and perceived reward contribute significantly to increased work engagement.

## 5. Discussion

In an increasingly dynamic and competitive organizational context, the role of telework has required new management approaches and more efficient strategies (Bentley et al., 2016), ensuring the sustainability and performance of organizations (Bilodeau et al., 2024; Pajor & Szeberényi, 2025). The perception of organizational support and the effectiveness of reward systems emerged as determinant factors that motivate and raise trust and work engagement (Gandrita et al., 2025). Several studies (e.g., Hong et al., 2023; Mehmood et al., 2023) have recognized that employee engagement is closely associated with positive organizational practices, particularly support and rewards. Therefore, examining how support and reward systems influence engagement and evaluating the mediating role of organizational trust becomes crucial for building more efficient work environments.

The results show that both POS and RS are positively related to WE, with RS having the greater direct effect. More specifically, in addition to the direct effects of POS and RS on WE, positive associations were found between POS and OT, RS and OT, and OT and WE. These findings suggest that POS and RS can predict OT and WE. It was also confirmed that OT is influenced by POS and RS, and correlates positively with WE. Moreover, the mediating role of OT between both variables and WE was verified, confirming its importance in the engagement process. In this study, all the relationships analyzed were validated, reinforcing the consistency of the hypotheses and the model proposed.

In addition to statistical evidence confirming the hypothesized relationships, the results are supported by Social Exchange Theory (SET), which posits that work relationships are characterized by reciprocal exchanges between employees and organizations. Consequently, employees who perceive organizational support, recognition, and fair treatment are more likely to respond by demonstrating greater trust in the organization and stronger engagement in their work (Hussain et al., 2026). As a result, employees are more dedicated, enthusiastic, and involved in their job roles (Sipayung & Nilasari, 2026).

In line with this theoretical perspective, the findings reinforce the central role of organizational trust in explaining employee engagement in the proposed model. Organizational trust emerged not only as a significant predictor of work engagement but also as a fundamental mediating mechanism through which perceived organizational support and reward systems influence employee attitudes and behaviors (Hong et al., 2023; Paillé et al., 2015). In telework contexts, trust acts as a crucial relational resource that consolidates the effects of organizational practices on employee engagement (Ha & Lee, 2022; Mehmood et al., 2023; Utomo et al., 2023). This evidence reinforces the relevance of organizational trust as a central mechanism in shaping positive work-related outcomes and corroborates the importance of fostering trust to enhance engagement and strengthen the effectiveness of human resource management practices (Hussain et al., 2026; Sipayung & Nilasari, 2026).

Considering the mediating role of organizational trust, it is essential to examine its main antecedents, particularly perceived organizational support, which constitutes an important driving factor in the formation of trust among employees. According to Eisenberger et al. (1986), high levels of POS reflect that the organization values the employees and prioritizes their well-being. It is based on this cognitive-affective process that workers develop positive perceptions of the organization, capturing organizational integrity and justice (Kurtessis et al., 2017; Seyrek & Çelik, 2025). For instance, some studies demonstrated that POS has a positive effect on trust and job satisfaction (Hasan et al., 2018), provides employees with indicators of recognition, increasing trust and positivity (Liang et al., 2023), and is a significant antecedent of organizational trust (Li et al., 2022). In teleworking contexts, the perception of organizational support is more relevant, as it can act as a relational compensation mechanism and an institutional indicator of reliability, promoting organizational trust.

In addition to organizational support, reward systems have also emerged as an important predictor of organizational trust in remote work environments. This finding is particularly consistent with Social Exchange Theory (Blau, 1986), which posits that when workers perceive benefits as fair (see organizational justice; Ha & Lee, 2022), they act reciprocally toward the company. Several studies attest to the benefits of reward policies as a differentiating resource in organizations that prioritize key psychosocial variables (Leitão et al., 2022; Mehmood et al., 2023; Ngamkroeckjoti et al., 2022). On the other hand, Saether (2020) and de Gagné and Deci (2014) asserted that poorly designed incentives lead to a significant reduction in employee trust, negatively affecting their dedication. According to Saether (2020), this occurs mainly because these mechanisms generate perceptions of inequality and injustice, as well as excessive control by the organization (Figueiredo et al., 2025).

Focusing on the role of organizational trust, the results suggest that it constitutes a fundamental mechanism for work engagement. Dirks and Ferrin (2002) showed that trust in leaders and the organization was associated with proactive attitudes, as well as greater productivity and organizational effectiveness. Thus, trust is understood not only as an individual psychological concept but also as a strategic resource. For instance, Wawrzynek (2023), when analyzing the formation of trust networks across different work models, concluded that low levels of trust in hybrid and remote contexts are due to decreased interactions. However, it is also important to highlight the disadvantages of compromised organizational trust, namely, a slowdown in the pace of work, which compromises productivity and performance. Thus, our findings indicate that organizations should promote OT, especially those that have offered a working-from-home option, because it is fundamental to adopt differentiating resources (e.g., performance recognition, opportunities for social interaction) that foster long-term trust (Wawrzynek, 2023).

Based on the structure of the proposed model, the POS analysis confirms its positive and direct effect on the work engagement of teleworkers, a finding that has been widely corroborated (e.g., Bilodeau et al., 2024; Eisenberger et al., 1986; Hussain et al., 2026; Khodakarami & Dirani, 2020; Sanhokwe, 2022; Selander & Alasoini, 2025). For instance, Bilodeau et al. (2024) concluded that this perception across remote work models does not result solely from institutional measures and policies, but is largely constructed through daily interactions between workers and supervisors. In teleworking, physical distance compromises social bonds and increases isolation (Figueiredo et al., 2025), which is why leadership plays a leading role. This recognition (e.g., feedback) influences the satisfaction of basic psychological needs and reveals the support provided by the organization. The perception of organizational support has become a phenomenon of particular interest in the context of remote work because it is strongly associated with work engagement. According to Selander and Alasoini (2025), high levels of support significantly increase engagement, even in the face of high cognitive demands or difficulties adapting to remote work. Contrary to what Lan et al. (2020) pointed out, several studies support the contribution of support to work engagement (e.g., Aldabbas et al. (2023); Mehmood et al. (2023)). These results highlight that employees who feel supported have more energy, are more resilient, more consistently committed to tasks, and demonstrate higher levels of concentration in their professional activities.

Finally, extending the analysis to the reward practices, the study also highlights their direct effect on work engagement, emphasizing the role as a key driver among remote workers. Several studies have analyzed this relationship (e.g., Figueiredo et al., 2025; Leitão et al., 2022; Nemțeanu et al., 2022; Ngamkroeckjoti et al., 2022; Pajor & Szeberényi, 2025; Tarigan et al., 2022) and concluded that reward practices are a strategic tool that enhances competitiveness. Gandrita et al. (2025) empirically demonstrated this dynamic, confirming that remote work is associated with higher levels of engagement and performance when reward systems are perceived as merit-based. In this context, valuing results and individual performance enhances the positive outcomes of teleworking, highlighting differentiating behavioral and organizational attitudes. Pajor and Szeberényi (2025) demonstrated that extrinsic rewards, particularly salary, were the most decisive factor for engagement. Reward systems, both financial and non-financial, play a critical role in enhancing the perception of organizational value and fairness. Although there is evidence strengthening the adverse effects of poorly designed incentives that lead employees to feel undervalued (e.g., Tarigan et al., 2022), authors such as Beqiri and Aziri (2022), Figueiredo et al. (2025), and Martono et al. (2018) reinforce that reward systems ensure positive results and sustainability for organizations.

### 5.1. Theoretical Implications

This study offers several theoretical contributions to the literature on human resource management, organizational behavior, and telework. First, it advances knowledge by empirically validating a model that links perceived organizational support, reward systems, organizational trust, and work engagement in a telework context. Validating this model helps clarify that engagement is strongly rooted in how employees feel treated, supported, and valued by their organization, especially when day-to-day interactions are mediated by technology rather than physical presence (Mäkikangas et al., 2022; Selander & Alasoini, 2025). While these constructs have been extensively examined in traditional, face-to-face work contexts, analysis in remote work settings remains limited (Sipayung & Nilasari, 2026). In addressing this gap, the study contributes to a more nuanced understanding of how different organizational resources operate when physical proximity and direct supervision are reduced.

Second, the findings extend Social Exchange Theory, demonstrating that its principles of reciprocity and mutual obligation remain highly applicable in working-from-home scenarios. The results show that teleworkers who perceive higher levels of support and fair reward systems are more likely to develop organizational trust, which is reflected in their engagement. This reinforces the idea that social exchange mechanisms are not weakened by physical distance, but can become even more salient when organizational resources are less visible and must be conveyed through formal practices and managerial actions.

Third, the study confirms the mediating role of organizational trust in the relationship between POS and reward systems and work engagement. By positioning organizational trust as a central explanatory mechanism, the study extends direct-effect models and clarifies how and why organizational practices translate into greater engagement among teleworkers. This mediating perspective enriches existing engagement models and highlights trust as a fundamental relational resource in current work systems. Finally, the findings provide evidence that classical constructs retain their explanatory power, interacting in a specific way in a telework context.

### 5.2. Practical Implications

The study offers practical implications for organizations that adopt or intend to expand telework. First, the findings highlight the strategic role of POS in maintaining employee engagement remotely. In remote work, employees rely more heavily on structured communication and messages from managers to feel valued and included (Sipayung & Nilasari, 2026). This means that organizations must ensure regular feedback, clear communication routines, and agile management support. Regular one-on-one virtual conversations and attention to workload and well-being can mitigate the absence of interactions (José et al., 2025). In addition, managers should be trained to adopt a supportive leadership style that combines autonomy and availability, ensuring that remote employees feel confident and guided (Buonomo et al., 2024). Second, the importance of reward systems on work engagement underscores the need for transparent, fair, and performance-aligned reward practices. Remote employees need to understand what is expected of them and how their contribution is assessed. At the same time, recognition should go beyond salary. Practices such as acknowledging good work in virtual meetings, offering flexible benefits, or making career opportunities more visible can help employees feel that their efforts are noticed, even remotely (Serhan et al., 2026). Non-monetary rewards such as autonomy, flexibility, and development opportunities become especially relevant and should be included in HR practices. Therefore, designing reward systems explicitly adapted to remote work can increase motivation, confidence, and continued engagement. Third, the mediating role of organizational trust suggests that organizational initiatives aimed at increasing engagement should not focus solely on isolated HR practices, but on building a broader climate of trust. While in office settings trust is built through face-to-face contact, manager behavior, and informal conversations, in telework, managers must establish clear communication, ensure consistency between promises and actions, and guarantee equitable and consistent treatment of all to build trust and increase the positive effects of support and rewards on engagement (Hussain et al., 2026). This also depends on giving employees real autonomy in organizing their work, while maintaining clear expectations. Hence, this study provides practical insights for organizational leaders and HR professionals, emphasizing that effective telework management depends not only on technological infrastructure but also on relational and psychosocial resources that foster long-term trust and engagement.

### 5.3. Limitations and Future Research Lines

This study is not without limitations that should be considered in future research. First, the use of a cross-sectional research design limits the conclusions and the ability to capture changes in perception, trust, and engagement over time. Future studies should adopt longitudinal designs to examine how these relationships evolve as telework regimes become more stable or hybrid. Second, the exclusive reliance on quantitative self-report measures may introduce a common method bias and does not provide a deeper exploration of subjective experiences. Future research could benefit from mixed-methods approaches to gain richer insights into teleworkers’ perceptions, motivations, and trust-building processes. Third, the study was conducted with teleworkers based in Portugal, which may limit the generalizability of the results to other cultural or institutional contexts. Cross-cultural studies or comparative research involving different countries and organizational contexts would help assess the robustness of the proposed model and identify potential contextual moderators. Furthermore, the study recruited teleworkers via e-mail and social media, enabling access to a diverse sample regarding sectors and types of organizations. However, it did not systematically collect detailed organizational variables such as company size, economic sector, or organizational origin, which consequently could not be included in the analysis. Future research should examine how these organizational characteristics may moderate the relationships under study, contributing to a more refined understanding of telework across different institutional contexts. Also, future research could expand the current model by incorporating additional variables, such as leadership style or the quality of digital communication. Exploring these factors could provide a more comprehensive understanding of the conditions under which organizational practices most effectively promote trust and engagement in remote work environments. Furthermore, since this study focuses exclusively on remote workers, it is not possible to conclude whether organizational trust plays a stronger, weaker, or equivalent role in employee engagement compared with on-site work settings. Future research using comparative or multi-group approaches will be valuable in directly addressing this gap and clarifying whether the observed relationships vary by work context.

More broadly, it would also be relevant to develop studies that more consistently integrate the structural and technological dimensions of telework, recognizing it as an evolving socio-technical phenomenon. In this sense, future research could examine how flexible and hybrid work arrangements reshape workers’ experiences in terms of autonomy, control, and well-being. Finally, given that these models may increase the demand for digital connectivity and intensify *telepressure* (Antunes et al., 2023; Barber et al., 2023), future studies could explore emerging issues such as the right to disconnect and new forms of pressure associated with digital work.

## 6. Conclusions

Teleworking has become an increasingly common practice and, as a result, there is a growing need to understand how organizational resources influence workers’ attitudes and behaviors when in-person interaction is reduced. Considering the role of psychological and contextual resources as determinants of performance and job satisfaction, this study explored the role of organizational perceptions and reward systems as strategic human resource management practices. In contexts characterized by supportive organizational policies and equitable incentives and recognition, teleworkers are more likely to perceive high-quality exchange relationships with their organization. From the perspective of Social Exchange Theory, these conditions foster reciprocity, leading remote workers to respond with confidence, greater commitment, and increased engagement. This study contributes to a deeper understanding of the relational dynamics between teleworkers and organizations, reinforcing that teleworking requires structured and intentional management practices. By investing in supportive policies, transparent reward mechanisms, and trust-building processes, organizations can foster confident, motivated, and engaged teleworkers. Consequently, the study provides relevant insights for organizations seeking to align HR management practices with employee expectations in remote work environments, supporting organizational sustainability and success.

## Figures and Tables

**Table 1:** Sociodemographic characterization.

Variable	N	%
Gender		
Female	224	55.3
Male	181	44.7
Age group		
22–25	98	24.2
26–35	217	53.6
36–45	20	4.9
45–53	70	17.3
Nationality		
Portuguese	402	99.3
Other	3	0.7
Marital status		
Single	250	61.7
Common-law union	40	9.9
Married	98	24.2
Divorced	17	4.2
Educational level		
Degree	190	46.9
Master	212	52.3
PhD	3	0.7
Seniority in the company		
[<1 year]	20	4.9
[1 to 5 years]	299	73.8
[5 to 10 years]	71	17.5
[>10 years]	15	3.7
Functions performed		
Administrative	31	7.7
Technical staff without managerial responsibilities	277	68.4
Technical staff with managerial responsibilities	96	23.7
Administration	1	0.2

**Table 2:** CFA and Cronbach’s Alpha analysis.

Latent Factor Construct/Factor	Items	CFA	α
Perceived Organizational Support Reward Systems Organizational Trust Work engagement	POS1 POS2 POS3 POS4 POS5 POS6 POS7 POS8 RS1 RS2 RS3 RS4 RS5 RS6 RS7 RS8 RS9 RS10 RS11 OT1 OT2 OT3 OT4 OT5 OT6 OT7 WE1 WE2 WE3 WE4 WE5 WE6 WE7 WE8 WE9	0.732 0.756 0.748 0.766 0.724 0.757 0.715 0.733 0.730 0.736 0.700 0.706 0.708 0.758 0.705 0.700 0.754 0.753 0.705 0.837 0.852 0.816 0.851 0.833 0.866 0.827 0.898 0.923 0.921 0.907 0.951 0.940 0.950 0.952 0.912	0.887 0.908 0.942 0.979

**Table 3:** Reliability and discriminant validity indices of the factors.

Factors	CR	AVE	POS	RS	OT	WE
POS	0.911	0.587	**0.766**			
RS	0.933	0.526	−0.171 ***	**0.725**		
OT	0.944	0.706	0.200 ***	0.247 ***	**0.840**	
WE	0.983	0.862	0.194 ***	0.531 ***	0.361 ***	**0.928**

Significance level: *** *p* < 0.001, CR: composite reliability; AVE: average variance extracted; bold values are the squared root of AVE, showing discriminant validity.

**Table 4:** Mean, standard deviation, and correlation matrix of all variables.

Variables	POS	RS	OT	WE	Gender	Age	Education	Role	Salary
POS	1								
RS	−0.171 **	1							
OT	0.200 **	0.247 **	1						
WE	0.194 **	0.531 **	0.361 **	1					
Gender	−0.025	0.030	−0.002	−0.007	1				
Age	−0.068	0.039	−0.007	0.048	0.021	1			
Education	−0.110 *	0.057	0.058	0.035	0.314 **	−0.145 **	1		
Role	−0.146 **	−0.062 **	−0.076 *	−0.047	−0.050	0.358 **	0.229 **	1	
Salary	−0.135 **	0.170 **	−0.047	0.035 **	0.020	0.150 **	0.533 **	0.311 **	1
Mean	2.99	3.12	3.30	3.32	1.553	31.87	3.538	3.168	4.758
SD	1.004	0.972	1.086	1.325	0.498	9.436	0.514	0.555	1.037

Significance level: ** *p* < 0.01. * *p* < 0.05.

**Table 5:** Model fit.

Fit Indices	Model Value	Reference Value
χ^2^/df	2.078	<3.00
CFI	0.948	>0.90
IFI	0.948	>0.90
TLI	0.945	>0.90
RMSEA	0.052	>0.08

**Table 6:** Hypothesis testing.

Path	Dimension	Dimension	β Estimate	SE	T
H1	POS →	OT	0.292	0.57	5,084 ***
H2	RS →	OT	0.342	0.58	5,939 ***
H3	OT →	WE	0.186	0.56	3,313 ***
H4	POS →	WE	0.393	0.63	6,275 ***
H5	RS →	WE	0.806	0.74	10,862 ***

Note: *** *p* < 0.01. SE: Standard Error.

## Data Availability

The raw data supporting the conclusions of this article will be made available by the authors upon request.
